# Erythema multiforme as first sign of incomplete Kawasaki disease

**DOI:** 10.1186/1824-7288-39-11

**Published:** 2013-02-13

**Authors:** Francesco Vierucci, Cristina Tuoni, Francesca Moscuzza, Giuseppe Saggese, Rita Consolini

**Affiliations:** 1 Pediatric Unit, Maternal & Infant Department, “S. Chiara” University-Hospital, Via Roma 67, Pisa 56126, Italy

**Keywords:** Incomplete Kawasaki disease, Erythema multiforme, Immunoglobulins

## Abstract

Incomplete Kawasaki disease represents a diagnostic challenge for pediatricians. In the absence of classical presentation, the laboratoristic evaluation of systemic inflammation can help in placing the correct diagnosis to promptly start adequate therapy. Erythema multiforme is an acute, self-limiting condition considered to be a hypersensitivity reaction commonly associated with various infections or medications. This aspecific skin condition has been rarely described as a sign of Kawasaki disease. We report on the case of a 4 years old boy presenting high-grade fever associated with erythema multiforme and evidence of systemic inflammation who showed a good response to prompt treatment with intravenous immunoglobulins.

## Background

Kawasaki disease is an acute, self-limited vasculitis of unknown etiology with a predilection for the involvement of coronary arteries, that affects predominantly infants and young children [1,2]. The diagnosis is based on the presence of fever associated with other transient typical signs, that rarely are simultaneously present at the time of first observation, but can appear subsequently. Indeed, there is no single pathognomonic clinical or laboratoristic finding for certain diagnosis. However, early recognition of Kawasaki disease is important to promptly start adequate therapy with intravenous immunoglobulins to prevent the development of coronary aneurysms [1,3-5]. Diagnosis of incomplete Kawasaki disease is even more difficult for pediatricians, because in the absence of classical presentation, vasculitis could be misdiagnosed and recognized late [6]; moreover, the incomplete form is at risk of heart complications, too [1,7]. Cutaneous manifestations are one of the diagnostic criteria in Kawasaki disease, but they are variable and non specific. Even if the typical findings of cutaneous changes are multiple symmetrical erythematous eruptions on the extensor surfaces of the extremities developing after 3–5 days of fever [1,8], Kawasaki disease may rarely present as erythema multiforme [9,10]. We report here on a case of a 4 years old boy with erythema multiforme as presenting sign of incomplete Kawasaki disease.

## Case presentation

**Figure 1:**
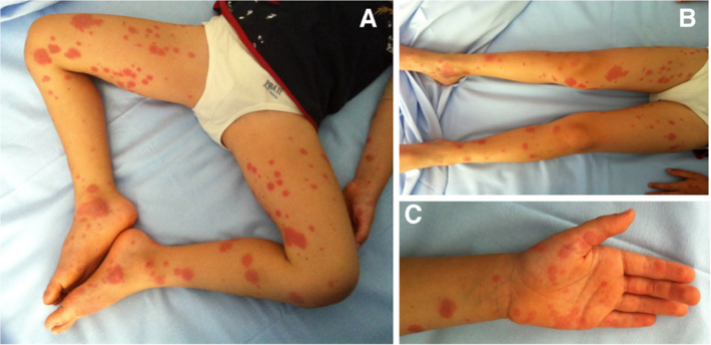
**Child’s cutaneous manifestations at hospital admission (2**^**nd **^**day of fever).** Lesions started acutely as numerous sharply demarcated red or pink macules that then became papular. Annular lesions were appreciable symmetrically on the distal extremities (**A** and **B**), involving also palms (**C**) and soles.

**Figure 2:**
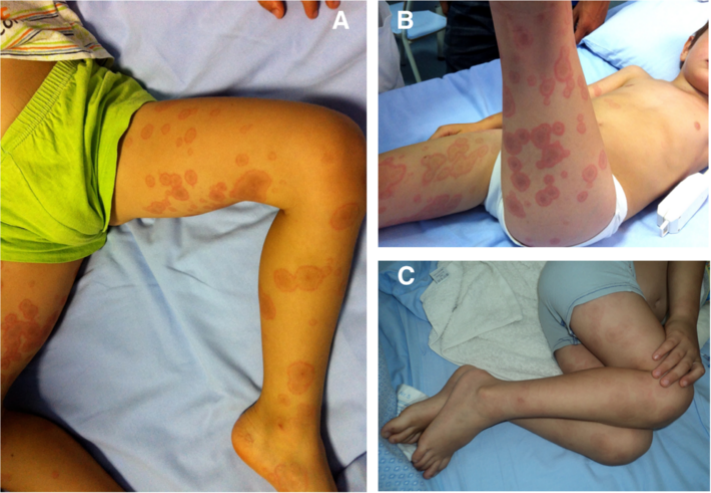
**Changes in child’s skin manifestations during hospitalization.** Annular lesions gradually enlarged into the characteristic “target” lesions with a regular round shape and three concentric zones: a central darker red area, a paler pink zone and a peripheral red ring. Figure shows skin lesions on 4^th^ day (**A**), 5^th^ day (**B**) of fever and the day after (7^th^ day) the administration of intravenous immunoglobulins (**C**).

A 4 years old boy was admitted to our Hospital for a one day history of remittent fever (up to 40.0°C), accompanied by irritability and annular, slightly itchy rash, started on his hands and feet and progressively extended to the flexor and extensor surfaces of the extremities, with relative sparing of the trunk (Figure 1). The child appeared extremely suffering. Physical examination showed bilateral lymphadenopathy (< 1.5 cm diameter) and hyperemic pharynx without exudate. The child did not report abdominal pain or arthralgia. Initial laboratoristic evaluation showed marked lymphocitosys with neutrophylia, hyponatremia and evidence of systemic inflammation (Table 1). As throat swab resulted positive for streptococcus pyogenes, parenteral administration of ceftriaxone was started. Infectious profile: blood and urine cultures, polymerase chain reaction for adenovirus, parvovirus B19, citomegalovirus, Epstein-Barr, virus herpes 6 virus, serology for herpes simplex virus, echovirus, coxsackie virus, mycoplasma pneumoniae were negative. Anti-nuclear antibody titer was negative. Abdomen ultrasound showed the absence of hepatosplenomegaly or hydrops of the gallbladder. Despite starting antibiotic therapy, the child persisted with remittent fever and irritability. Annular cutaneous manifestations evolved to multiple target-like erythematous lesions compatible with erythema multiforme (Figure 2A and B). Blood test performed in 4^th^ day of fever confirmed the picture of systemic inflammation (Table 1). In 6^th^ day of fever the child showed mild bilateral bulbar conjunctival injection without exudate. Elevated antistreptolysin O antibody titer confirmed recent streptococcus pyogenes infection. Electrocardiogram revealed abnormalities in ventricular repolarization (T-waves negative in V6), but echocardiography did not show coronary alterations.

**Table 1:** Laboratoristic evaluation during hospitalization and follow-up

**Investigation**		**2**^**nd **^**day admission**	**4**^**th**^ day	**6**^**th **^**day IVIg**	**8**^**th **^**day**	**10**^**th **^**day**	**12**^**th **^**day discharge**	**26**^**th **^**day**
White blood cell count	mm^3^	18 400	17 140	10 210	3 960	4 590	4 410	4 350
Red blood cell count	mm^3^	5 010 000	4 550 000	3 760 000	4 390 000	4 200 000	4 410 000	4 330 000
Hemoglobin	g/dl	13.6	12.5	10.2	12.1	11.4	11.9	12.1
Platelets	mm^3^	262 000	211 000	231 000	380 000	498 000	543 000	210 000
Neutrophils	%	88.5	89.5	79.4	43.7	28.7	43.1	29.4
ESR	mm/h		62	64		85	78	19
CRP	mg/dl	9.21	17.36	12.94	4.88	2.11	0.89	0.02
Procalcitonin	ng/ml	3.31	6.74		0.71	0.31		0.05
Albumin	g/dl		3.4	3.0				
Sodium	mEq/l	131	134	133	132		137	
Potassium	mEq/l	4.52	3.47	2.77			4.48	
AST	U/l	29	65	46			39	
ALT	U/l	15	20	18		19	19	
GGT	U/l	13	13	9		11	13	
Fibrinogen	mg/dl	425	560					
Ferritin	ng/ml			399				
Antistreptolysin O titer	U/ml			3 460				
Urine white blood cells	mm^3^	8		126		2	0	

IVIg: intravenous immunoglobulins.

ESR: erythrocyte sedimentation rate.

CRP: C-reactive protein.

AST: aspartate aminotransferase.

ALT: alanine aminotransferasi.

GGT: gamma-glutamyl transpeptidase.

Diagnosis of incomplete Kawasaki disease was posed on the basis of the presence of fever persisting at least 5 days, associated to 2 classic diagnostic criteria (polymorphous exanthem and aseptic conjunctival injection), increased levels of ESR and CRP with 4 supplemental laboratory criteria (hypoalbuminemia, anemia, leucocytosis and leucocyturia).

Treatment with intravenous immunoglobulins (2 gr/Kg) and high-dose aspirin was promptly started. After immunoglobulins administration the child's clinical conditions improved with defervescence and reduction in systemic inflammation indexes. After 24 hours the child presented again transient fever up to 39.5°C, that responded to paracetamol with final defervescence. Cutaneous lesions progressively faded (Figure 2C). Aspirin dose was reduced to low-dose (5 mg/Kg per day), after the child has been afebrile for 48 hours. As expected, on 10^th^ day blood analysis showed thrombocytosis. On 12^th^ day from the onset of the fever electrocardiogram and cardiac ultrasound were normal, so the child was discharged. Two weeks after the discharge erythema multiforme was completely resolved, no desquamation was observed and flogosis indexes returned into normal range. Low-dose aspirin was maintained until patient shows no evidence of coronary changes by 8 weeks after the onset of illness. Cardiologic follow-up in the next 6 months was normal.

## Discussion

Kawasaki disease is the most common systemic vasculitis in childhood after Henoch-Schonlein purpura and the most common cause of acquired heart disease among children living in West countries [11]. Since its identification in 1967 [12], it is now accepted that Kawasaki disease can be considered as a continuous spectrum, ranging from incomplete to complete forms, that include all diagnostic criteria [13]. Diagnostic criteria of Kawasaki disease postulated by the American Heart Association are fever persisting at least 5 days in presence of at least 4 principal features such as changes in extremities (acute erythema of palms/soles or edema of hands/feet; subacute periungual peeling of fingers/toes), polymorphous exanthem, bilateral bulbar conjunctival injection without exudate, changes in lips and oral cavity (erythema, lips cracking, strawberry tongue, diffuse injection of oral and pharyngeal mucosa), cervical lymphadenopathy (> 1.5 cm diameter), usually unilateral [1]. All these signs are non specific and frequently overlap with other diseases, particularly with systemic juvenile idiopathic arthritis [1]. In our case, this condition was excluded for the absence of typical clinical features such as arthritis, intermittent fever, evanescent erythematosus rash, hepatosplenomegaly or serositis. The prevalence of incomplete Kawasaki disease is 15-36% [6]. A recent report of a series of 955 patients showed that the cases presenting the incomplete form represented the 23% of the whole cohort; patients with incomplete Kawasaki disease had a 1 day longer median interval from onset to diagnosis and were less likely to be treated with intravenous immunoglobulins (86% versus 96%). However, overlapping laboratory findings and coronary artery abnormalities confirmed that the two forms can be considered as two sides of the same coin [7]. Incomplete Kawasaki disease should be considered in all children with unexplained fever for ≥ 5 days associated with 2 or 3 of the principal clinical features of Kawasaki disease, according to the algorithm proposed by American Heart Association [1]. Laboratory findings can help in diagnosis if systemic inflammation (ESR ≥ 40 mm/hr and CRP ≥ 3.0 mg/dL) is associated to other supplemental laboratory criteria such as hypoalbuminemia (albumin < 3.0 g/dL), anemia for age, elevation of alanine aminotransferase, trombocythosis (platelets after 7 days > 450 000/mm^3^), leucocytosis (white blood cell count > 15 000/mm^3^) and sterile leucocyturia (urine > 10 white blood cells/high-power field). In presence of at least 3 supplemental criteria, as in our case, treatment with intravenous immunoglobulins and cardiac ultrasound have to be performed [1].

The case we reported is particularly interesting for the unusual presentation of incomplete Kawasaki disease. Indeed, erythema multiforme was reported as a cutaneous manifestation of classic Kawasaki disease in only 2 young children, a 22-month-old girl in 1979 [9] and a 16-month-old boy in 2010 [10]. In addition, other 3 patients were described affected by Kawasaki disease associated to annular lesions [14]. To our knowledge, this is the first report of erythema multiforme as first sign of incomplete Kawasaki disease.

The skin eruption of Kawasaki disease has been described as an erythematous rash usually appearing within 5 days of the onset of fever. The most common is a non specific, diffuse maculopapular eruption. Occasionally some other skin pictures such as urticarial exanthem, scarlatiniform rash, erythroderma, erythema-multiforme-like rash or, rarely, fine micropustular eruption have been described. The rash usually is extensive, with involvement of the trunk and extremities and accentuation in the perineal region, where early desquamation may occur [1]. In our case the rash erupted the first day of fever as annular, slightly itchy cutaneous manifestations that evolved to multiple target-like erythematous lesions compatible with erythema multiforme in 4^th^ day of fever. Erythema multiforme was more appreciable at the extremities, including palms and soles, with relative unusual sparing of the trunk. We did not observed changes in the extremities, neither changes of the lips and oral cavity.

Erythema multiforme is an acute, self-limited, sometimes recurring, skin condition considered to be a hypersensitivity reaction associated with certain infections, particularly herpes simplex virus and mycoplasma pneumoniae, and with medications such as penicillins and non steroidal anti-inflammatory drugs, commonly used in children [15]. It usually occurs in adults 20 to 40 years of age, although it can occur in children [15,16]. Erythema multiforme usually has mild or no prodromal symptoms, and patients may experience itching and burning at the site of the eruption. Typical target lesions may not be apparent until several days after the onset, when lesions of various clinical morphology usually are present, hence the name erythema “multiforme” [15,17]. Diagnosis of erythema multiforme is clinical, and requires careful differential diagnosis because its presentation is associated to an extremely wide series of diseases, such as drug eruption, lupus erythematosus, Stevens-Johnson syndrome, toxic epidermal necrolysis, urticaria, viral exanthems and vasculitis [15].

In the case we described there was no development of coronary abnormalities, but electrocardiography showed completely reversible abnormalities in ventricular repolarization. This finding is compatible with Kawasaki disease, where ECG may show arrhythmia, prolonged PR interval, or non specific ST and T wave changes [1]. Finally, we demonstrated previous streptococcus pyogenes infection and we hypothesized it as a trigger for Kawasaki disease development, as previously described [14,18]. An infectious trigger of Kawasaki disease has been suspected by the epidemiologic features, such as age of affected children, seasonality of cases, and occurrence of community outbreaks and epidemics [1]; however, no known infectious agent has been consistently found [19]. As streptococci are infectious agents associated even with erythema multiforme [20], therefore it is possible to speculate that, in our case, streptococcus pyogenes infection may have been a common trigger underlying both conditions, Kawasaki disease being the end result of an extensive immune activation in a predisposed host.

After the publication of American Heart Association algorithm in 2004, the diagnosis of incomplete Kawasaki disease is increased, primarily due to a systematic performance of laboratoristic evaluation [21]. Indeed, even if the laboratory findings of incomplete cases appear to overlap to those of classic ones, they may prove useful in heightening or reducing the suspicion of incomplete Kawasaki disease [1]. Nevertheless, the description of Kawasaki disease cases, characterized by unusual presentation, in terms of clinical signs, is extremely useful to plan the diagnosis of a so intriguing disease.

## Conclusions

The paper indicates erythema multiforme as a possible early cutaneous manifestation of Kawasaki disease, particularly in the case of the incomplete form. This acquisition is strongly important in the pediatric practice to avoid delaying in diagnosis and to promptly start adequate treatment.

## Consent

Written consent was obtained from the patient's parents for publication of this case report and accompanying images. A copy of the written consent is available for review by the Editor-in-Chief of this journal.

## Competing interests

The authors declare that they have no competing interests.

## Authors’ contribution

FV: prepared the manuscript and search the literature. CT: prepared the manuscript. FM: prepared the manuscript. GS: reviewed the manuscript. RC: made the diagnosis and reviewed the manuscript. All authors read and approved the final manuscript.
